# Dose‐Dependent Biphasic Effect of Palmitic Acid on Oligodendrocyte Function: Impacts on Viability, Differentiation, and Myelination

**DOI:** 10.1002/jcp.70145

**Published:** 2026-02-09

**Authors:** Anna Palmiero, Luca Pipicelli, Giuliana La Rosa, Concetta Sozio, Carolina Punziano, Maddalena Raia, Raffaella Faraonio, Giovanna Vitolo, Mariarosaria Cammarota, Francesca Boscia, Ciro Menale, Mariarosaria Santillo, Simona Damiano

**Affiliations:** ^1^ Department of Clinical Medicine and Surgery University of Naples “Federico II” Naples Italy; ^2^ Department of Molecular Medicine and Medical Biotechnology University of Naples “Federico II” Naples Italy; ^3^ CEINGE‐Biotecnologie Avanzate “Franco Salvatore” Naples Italy; ^4^ Department of Neuroscience, Reproductive Sciences and Dentistry, School of Medicine “Federico II” University of Naples Naples Italy

**Keywords:** differentiation, mitochondrial dynamics, multiple sclerosis, myelination, Nrf2, oligodendrocyte, palmitic acid

## Abstract

Palmitic acid (PA), the most abundant saturated fatty acid (SFA) in humans, plays a key role in energy metabolism, membrane synthesis, and signaling. Oligodendrocyte precursor cells (OPCs), which generate mature oligodendrocytes (OLs) forming the myelin sheath, are responsive to metabolic and redox signals. Despite increasing interest in lipid metabolism and mitochondrial dynamics as regulators of OPC fate, the effects of PA remain unclear. This study investigates the biphasic, dose‐dependent effects of PA on OPCs using the oligodendrocyte precursor MO3.13 cell line and employs rat organotypic slice cultures to evaluate the effects of non‐toxic PA doses under pathological conditions and on axonal (re)‐myelination. In MO3.13 cells, high‐dose PA (100 µM) induces mitochondrial fragmentation and caspase‐7 activation, accompanied by reduced mitofusin‐2 (MFN2) and phosphorylated dynamin‐related protein 1 at Ser616 (p‐DRP1), indicating altered fusion‐fission balance and impaired reactive oxygen species (ROS) generation. In contrast, low‐dose PA (25 µM) triggers a protective response involving nuclear factor erythroid 2–related factor 2 (Nrf2) activation and upregulation of antioxidant and lipid‐regulatory genes (glutamate–cysteine ligase modifier subunit [GCLM], NAD(P)H dehydrogenase [quinone] 1 [NQO1], peroxisome proliferator‐activated receptor gamma [PPARγ], and cluster of differentiation 36 [CD36]) resulting in reduced intracellular ROS and enhanced lipid mobilization. PA 25 µM promotes OPC differentiation by inhibiting migration and cell cycle progression and increasing myelin basic protein (MBP) and proteolipid protein (PLP) expression. Notably, early exposure (1 day) favors mitochondrial fusion, whereas prolonged exposure (4 days) shows a physiological shift to fission. PA 25 µM prevents neurodegeneration in hippocampal organotypic slice cultures exposed to a neuroinflammatory insult. In cerebellar organotypic slice cultures, PA 25 µM enhances axonal myelination and accelerates remyelination following lysolecithin‐induced demyelination. These findings highlight the physiological relevance of low‐dose PA in modulating OLs.

## Graphical abstract

Schematic diagram showing dose‐dependent effects of PA in oligodendrocyte precursor MO3.13 cells and rodent organotypic hippocampal and cerebellar slice cultures. High doses lead to cell death accompanied by mitochondrial impairment, whereas low doses activate the Nrf2 antioxidant response and exert neuroprotective effect while balancing mitochondrial dynamics and promoting cellular differentiation and axonal myelination.

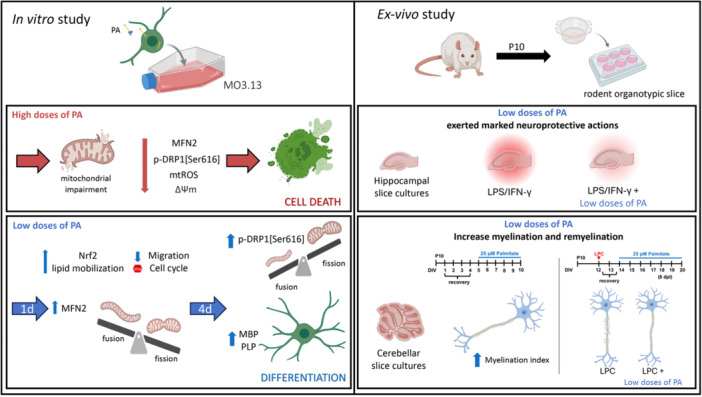

**Abbreviations**
AR: aspect ratio
AXV: annexin V
BBB: blood–brain barrier
BSA: bovine serum albumin
CA1: cornu ammonis area 1
CaCl_2_
: calcium chloride
CD36: cluster of differentiation 36
cDNA: complementary DNA
CNS: central nervous system
CO_2_
: carbon dioxide
DAPI: 4′,6‐diamidino‐2‐phenylindole
DCFH‐DA: 2′,7′‐dichlorodihydrofluorescein diacetate
DHE: dihydroethidium
DIV: days in vitro
DMEM: Dulbecco's modified eagle medium
DMSO: dimethyl sulfoxide
DNL: de novo lipogenesis
DOC: sodium deoxycholate
DPL: day postlysolecithin
DRP1: dynamin‐related protein 1
DTT: dithiothreitol
ECL: enhanced chemiluminescence
EDTA: ethylenediaminetetraacetic acid
FA: fatty acid
FBS: fetal bovine serum
FF: form factor
GAPDH: glyceraldehyde 3‐phosphate dehydrogenase
GCLM: glutamate–cysteine ligase modifier subunit
HBSS: Hank's balanced salt solution
HEPES: 4‐(2‐hydroxyethyl)‐1‐piperazineethanesulfonic acid
HRP: horseradish peroxidase
IF: immunofluorescence
IFN‐γ: interferon gamma
KCl: potassium chloride
LD: lipid droplet
LPC: lysophosphatidylcholine
LPS: lipopolysaccharide
MBP: myelin basic protein
MFN2: mitofusin‐2
MgCl_2_
: magnesium chloride
MO3.13: human oligodendrocyte cell line MO3.13
MS: multiple sclerosis
mtROS: mitochondrial reactive oxygen species
MTT: 3‐(4,5‐dimethylthiazol‐2‐yl)‐2,5‐diphenyltetrazolium bromide
NaCl: sodium chloride
NaF: sodium fluoride
NaPPi: sodium pyrophosphate
NaVO_4_
: sodium orthovanadate
NF‐κB: nuclear factor kappa‐light‐chain‐enhancer of activated B cells
NQO1: NAD(P)H dehydrogenase [quinone] 1
Nrf2: nuclear factor erythroid 2‐related factor 2
OPCs: oligodendrocyte precursor cells
PA: palmitic acid
PBS: phosphate‐buffered saline
PEA: palmitoylethanolamide
PFA: paraformaldehyde
PI: propidium iodide
PIC3: phosphatase inhibitor cocktail III
PIs: protease Inhibitors
PLP: proteolipid protein
PMA: phorbol 12‐myristate 13‐acetate
PMSF: phenylmethylsulfonyl fluoride
PPARγ: peroxisome proliferator‐activated receptor gamma
PUFAs: polyunsaturated fatty acids
p‐DRP1[Ser616]: phosphorylated dynamin‐related protein 1 at Ser616
qPCR: quantitative polymerase chain reaction
RNA: ribonucleic acid
RNase A: ribonuclease A
ROS: reactive oxygen species
SDS: sodium dodecyl sulfate
SEM: standard error of the mean
SFA: saturated fatty acid
TBS‐T: tris‐buffered saline with Tween 20
TLR: toll‐like receptors
TMRE: tetramethylrhodamine, ethyl ester
Tris‐HCl: tris(hydroxymethyl)aminomethane Hydrochloride
β2M: beta‐2 microglobulin
ΔΨm: mitochondrial membrane potential

## 1 Introduction

Oligodendrocytes (OLs) are the myelin forming cells of the central nervous system (CNS). Myelin sheath is a lipid‐rich structure that enwraps neuronal axons enabling rapid transmission of electrical signals. In addition to their myelinating role, OLs provide trophic support to neurons contributing to their maintenance and survival (Philips and Rothstein 2017). Myelinating OLs arise from oligodendrocyte progenitor cells (OPCs) (Trotter et al. 2010; Beiter et al. 2022) through a multistep maturation process comprising migration, proliferation, and differentiation (Emery and Wood 2024), ensuring myelin turnover and replacement in demyelinating diseases, such as multiple sclerosis (MS), where a differentiation impairment has been documented in both human tissues and experimental models (Tepavčević and Lubetzki 2022; Kuhlmann et al. 2008). OL differentiation critically depends on mitochondrial function, and mitochondrial dysfunction has been implicated in impaired OL maturation, defective myelin production, and increased susceptibility to oxidative stress (Bame and Hill 2024). In this context, mitochondrial quality control mechanisms play a pivotal role in preserving mitochondrial integrity and guiding cell fate decisions during OL differentiation and myelin formation (Bame and Hill 2024). Myelination is an energy‐intensive process that requires a continuous supply of fatty acids (FA), which represent key structural components of myelin and whose availability is sustained by mitochondrial‐dependent metabolic pathways (Poitelon et al. 2020). Among FAs, palmitic acid (PA), the most abundant saturated long‐chain FA in mammals, plays a central role as a precursor for membrane lipids and other bioactive molecules (Smith and Bazinet 2024).

PA can be endogenously synthesized through de novo lipogenesis (DNL) (Carta et al. 2017), a process enhanced by high‐carbohydrate or high‐fat diets (Murru et al. 2022). In addition to serving as an energy substrate, PA influences membrane properties and regulates protein localization and function through palmitoylation (Carta et al. 2017). PA also supports the synthesis of bioactive lipids such as palmitoylethanolamide (PEA), whose production increases under cellular stress as part of protective responses. Additionally, recent studies have highlighted the ability of this bioactive molecule to promote the differentiation of OLs (Valenza et al. 2022). Some studies also report a negative correlation between PA levels and MS‐related disability (Hon et al. 2009). Despite its physiological relevance, PA accumulation can exert detrimental effects when present at excessive concentrations. Increased PA availability, resulting from altered lipid metabolism or enhanced de novo lipogenesis, has been associated with lipotoxicity, oxidative stress, and mitochondrial dysfunction in multiple cell types (Langley et al. 2020; Rachek et al. 2007; Engin 2017).

Moreover, in the CNS, high PA levels have been linked to neuroinflammatory signaling, (Vesga‐Jiménez et al. 2022). Indeed, in neuroinflammatory conditions, FA metabolism is profoundly reshaped, shifting toward increased synthesis and accumulation of pro‐inflammatory saturated and ω−6 fatty acids such as palmitic and arachidonic acid, while anti‐inflammatory ω−3 polyunsaturated fatty acids (ω−3 PUFAs) decline (Calder 2012; Farooqui et al. 2007). This imbalance can promote inflammatory mediators and reactive oxygen species (ROS) production. Activation of the transcription factor nuclear factor erythroid 2–related factor 2 (Nrf2) represents a key adaptive response that promotes antioxidant and cytoprotective gene expression, thereby contributing to cellular resilience against oxidative and metabolic insults (Ma 2013). PA has been reported to promote a pro‐inflammatory microenvironment that can compromise blood‐brain barrier (BBB) integrity; once the BBB is disrupted, PA penetrates the brain, causing neurotoxic effects on neurons and glial cells (Patil et al. 2007; Freitas et al. 2017; Kim et al. 2016; Rhea et al. 2017). This is particularly relevant in MS, where BBB disruption allows immune cell infiltration, leading to demyelination (Niu et al. 2019). The dual beneficial and detrimental effects of PA reveal its complex role in disease progression. Nevertheless, the mechanisms by which PA affects OLs, particularly in relation to mitochondrial homeostasis and differentiation, remain poorly understood. Using the immortalized human oligodendrocyte precursor cell line MO3.13, this study investigates the biphasic dose‐dependent effects of PA on OL biology. Furthermore, the protective and re‐myelinating actions of low dose PA were investigated in three‐dimensional (3D) tissue‐like CNS models of neuroinflammatory degeneration and myelin damage and repair. By elucidating the dual role of PA in regulating mitochondrial homeostasis and OL differentiation, this work aims to provide new insights into lipid‐mediated mechanisms influencing myelination and remyelination in the CNS.

## 2 Materials and Methods

### 2.1 Palmitic Acid‐BSA Conjugated Preparation

Palmitic acid (PA; Sigma‐Aldrich, St. Louis, MO, USA) was prepared using FA‐free BSA (Sigma‐Aldrich, St. Louis, MO, USA) as the vehicle, by dissolving a 16 mM palmitate solution in 150 mM NaCl at 70°C and preparing a 10% (1.5 mM) FA‐free BSA solution in the same buffer. These solutions were then mixed at a 1:1 ratio to obtain a final concentration of 8 mM PA conjugated to 5% FA‐free BSA. The final molar ratio of PA to BSA was 10:1.

### 2.2 Cell Cultures and Treatments

The immortal human‐human hybrid cell line MO3.13 (CELLution Biosystem Inc., Toronto, ON, Canada; cat# CLU301; RRID: CVCL_D357) was derived from the fusion of a 6‐thioguanine‐resistant mutant of a human rhabdomyosarcoma with oligodendrocytes obtained from an adult human brain. This cell line was free of mycoplasma contamination. Cells were cultured in Dulbecco's Modified Eagle's Medium (DMEM), containing 4.5 g/L glucose (GIBCO, Thermo Fisher Scientific, Waltham, MA, USA) and l‐glutamine 0.584 g/L, supplemented with 10% fetal bovine serum (FBS; Gibco, Thermo Fisher Scientific, Waltham, MA, USA), 100 U/mL penicillin and 100 µg/mL streptomycin. MO3.13 cells were differentiated using a differentiation medium consisting of FBS‐free DMEM supplemented with 100 nM Phorbol 12‐myristate 13‐acetate (PMA) (Sigma‐Aldrich, St. Louis, MO, USA) (Damato et al. 2021; Ursell et al. 1995; Głowacka et al. 2022). The medium was replaced every 2 days. Cells were cultured in a humidified atmosphere of 5% CO₂ and 95% air at 37°C.

### 2.3 Cell Viability

Viability of MO3.13 cells upon different concentrations of PA was evaluated. Briefly, 2 × 10^4^ cells were plated in 24‐well plates in complete DMEM; after 18 h, cells were treated for 24 h with increasing doses of PA from 12.5 µM to 800 µM and counted through the Trypan blue dye‐exclusion method to assess the cytotoxicity of PA. Specifically, after trypsinization and washing in phosphate‐buffered saline (PBS), the cells were resuspended in diluted Trypan blue and then immediately counted in a Bürker chamber. The viable (unstained) and unviable (stained) cells were counted separately. The percentage of viable cells was calculated as follows:

CellViability(%)=[1−(numberofnon−viablecells/totalnumberofcells)]×100

Gross mitochondrial metabolic activity of the cells under the previously described conditions was evaluated using the MTT (3‐(4,5‐dimethylthiazol‐2‐yl)−2,5‐diphenyltetrazolium bromide) colorimetric assay (Sigma‐Aldrich, St. Louis, MO, USA). This assay measures the activity of enzymes that reduce MTT to formazan, producing blue/purple crystals that precipitate within the cells. This reduction primarily occurs in mitochondria through the action of succinate dehydrogenase, an enzyme active in viable and metabolically active cells. The enzyme cleaves the tetrazole ring of MTT (a yellow substance), resulting in the formation of formazan (a blue salt). Specifically, 1 × 10⁴ cells were seeded in 96‐well plates and incubated for 24 h in medium containing 0.2% FBS, in the presence or absence of PA and BSA (vehicle). Following incubation, the cells were treated with MTT solution (0.5 mg/mL) for 2 h at 37°C. After incubation, the formazan salts were dissolved in a DMSO/Isopropanol (1:1, v/v) solution and the reaction product was quantified using spectrophotometric measurement at a wavelength of 595 nm.

### 2.4 Mitochondrial Morphology and Membrane Potential Analysis

Mitochondrial morphology was assessed using the MitoTracker Red CMXRos dye (Invitrogen, Thermo Fisher Scientific, Waltham, MA, USA). This probe passively diffuses across the plasma membrane and accumulates in the mitochondria of live cells. In brief, MO3.13 cells were cultured on glass coverslips and incubated with 50 nM MitoTracker Red CMXRos in DMEM at 37°C for 20 min in the dark. After incubation, the cells were fixed with 3.7% paraformaldehyde (PFA) and counterstained with 4′,6‐diamidino‐2‐phenylindole (DAPI; 1 μg/mL, Sigma‐Aldrich, St. Louis, MO, USA) to visualize nuclei. Images were acquired using a Leica DMi8 fluorescence microscope equipped with the Leica Application Suite (LAS X) imaging software. Mitochondrial morphology was analyzed using ImageJ software (NIH, Bethesda, MD, USA) with the Mitochondria Analyzer plug‐in (Menale et al. 2023). Values were determined by analyzing 50 cells per sample from at least three independent experiments performed in triplicate.

Mitochondrial membrane potential (ΔΨm) was assessed using the TMRE (tetramethylrhodamine ethyl ester, Invitrogen, Thermo Fisher Scientific, Waltham, MA, USA) fluorescent probe. In brief, MO3.13 cells were resuspended in pre‐warmed DMEM and incubated with 200 nM TMRE at 37°C for 30 min in the dark. After staining, cells were washed twice with PBS to remove excess dye and immediately analyzed. TMRE fluorescence was measured using a BD FACSCanto™ II flow cytometer (BD Biosciences, Franklin Lakes, NJ, USA), collecting at least 50,000 events per sample. Data were analyzed using FlowJo software (BD Biosciences, Franklin Lakes, NJ, USA), and ΔΨm values were expressed relative to control cells. Experiments were performed in triplicate from at least three independent biological replicates.

### 2.5 Reactive Oxygen Species (ROS) Analysis

The effect of PA on mitochondrial ROS was measured with MitoSOX Red (1 µM; Invitrogen, Molecular Probes, Thermo Fisher Scientific, Waltham, MA, USA), a specific indicator of mitochondrial superoxide (excitation/emission 510/580 nm). We used 1 µM because at 5 µM is not specific to mitochondria and can lead to cellular dysfunction due to mitochondrial overload (Roelofs et al. 2015). The effect of PA on intracellular ROS in MO3.13 cells was also evaluated using the fluorescent probe dihydroethidium (DHE; 10 µM; Invitrogen, Molecular Probes, Thermo Fisher Scientific, Waltham, MA, USA) (excitation/emission 500/580 nm). Briefly, MO3.13 were cultured on glass coverslips in complete DMEM medium. The following day, the complete culture medium was removed and medium containing 0.2% FBS was added for 24 h and treated with different concentrations of PA (Sigma‐Aldrich, St. Louis, MO, USA). Then, cells were fixed in 3.7% PFA for 15 min at room temperature. Nuclei were stained with DAPI 1 µg/mL (Sigma‐Aldrich, St. Louis, MO, USA). Images were acquired using a Leica DMi8 fluorescence microscope with a 20x objective and analyzed using ImageJ software (National Institutes of Health, Bethesda, MD, USA) following the method described by McCloy et al (La Rosa et al. 2023; McCloy et al. 2014).

Then intracellular ROS levels in MO3.13 were evaluated using the membrane‐permeant ROS sensitive fluorogenic probe 5,6‐carboxy‐2, 7‐dichlorofluoresceindiacetate, DCHF‐DA (excitation/emission 495/520 nm) (Sigma‐Aldrich, St. Louis, MO, USA) (10 µM). Specifically, 2 × 10^4^ cells were counted and grown in a 24‐well plate in complete DMEM as previously described. Cells were washed in PBS containing 10 mM glucose, 1.2 mM MgCl_2_ and 1.2 mM CaCl_2_ and dichlorofluorescein (DCF) fluorescence was measured at different time intervals, using the plate reader Fluoroskan Ascent FL fluorometer (Thermo Electron Oy, Vantaa, Finland), and analyzed by Ascent software (v 2.4).

### 2.6 Cell Apoptosis Evaluation

The apoptosis level of MO3.13 cells was quantified with the dead cell apoptosis assay with Annexin V Alexa Fluor 488 (AXV; Thermo Fisher Scientific, Waltham, MA, USA) and Propidium Iodide (PI; Thermo Fisher Scientific, Waltham, MA, USA) staining according to the manufacturer's instructions. Specifically, PI can enter necrotic cells due to compromised membrane integrity and binds to DNA, whereas annexin V binds to phosphorylated serine on the outer leaflet of plasma membrane in apoptotic cells. In brief, MO3.13 cells were grown to semi‐confluence in 6 multiwell plates and next treated for 24 h medium containing 0.2% FBS, with PA (25 µM, 100 µM), then cells were incubated in Annexin V binding‐buffer (50 mM HEPES, 700 mM NaCl, 12.5 mM CaCl_2_, pH 7.4) containing Alexa Fluor 488 Annexin V and with PI before reading for cytometry analysis using FACSCanto II flow cytometer (Becton Dickinson, BD Bioscience, Franklin Lakes, NJ, USA). Data were analyzed by FlowJo software v.10 (BD Life Sciences). Early apoptotic cells were identified by quantifying the percentage of AXV^+^/PI^+^ cells, while necrotic cells were detected based on the percentage of AXV^‐^/PI^+^ cells. Negative cells for both AXV and PI (AXV^‐^/PI^‐^) were classified as viable.

### 2.7 Protein Extraction

MO3.13 cells were grown to semi‐confluently in 6‐well plates in complete DMEM, then starved for 18 h in medium containing 0.2% FBS, and treated with PA (25 and 100 μM) or differentiation medium.

Total protein lysates were extracted using RIPA buffer containing 50 mM Tris(hydroxymethyl)aminomethane hydrochloride (Tris‐HCl; pH 7.5), 150 mM sodium chloride NaCl, 1% NP‐40, 0.5% sodium deoxycholate (DOC), 0.1% sodium dodecyl sulfate (SDS), 2.5 mM Na‐pyrophosphate (NaPPi), 1 mM β‐glycerophosphate (β‐GP), 1 mM sodium orthovanadate (NaVO₄), 1 mM sodium fluoride (NaF), 0.5 mM phenylmethylsulfonyl fluoride (PMSF), a 1X protease inhibitors (PIs) cocktail (Roche Applied Bioscience, Penzberg, Germany) and a phosphatase inhibitor cocktail III (PIC3; Sigma‐Aldrich, St. Louis, MO, USA).

Cells were lysed mechanically by repeated aspirations through a needle and incubated on ice for 10 min. The lysates were then centrifuged at 15,000 × *g* for 10 min at 4°C.

Extracts of nuclear and cytosolic proteins were obtained using two different buffers: Buffer A for cytosolic proteins and Buffer B for nuclear proteins.

For cytosolic protein extraction, cells were incubated with 60 µL of buffer A (contained 10 mM 4‐(2‐hydroxyethyl)−1‐piperazineethanesulfonic acid (HEPES), 1.5 mM magnesium chloride (MgCl₂), 10 mM potassium chloride (KCl), 0.5 mM dithiothreitol (DTT), 0.05% NP‐40, 1X PIs, 574 μM phenylmethylsulfonyl fluoride (PMSF), 1 mM sodium orthovanadate (NaVO₄), 1 mM sodium fluoride (NaF), 2.5 mM NaPPi, and 1 mM β‐GP, pipetted up and down 10 times and placed on ice for 10 min. The samples were then centrifuged at 800 × *g* for 10 min at 4°C. The supernatant, containing cytosolic proteins, was collected. The pellet was washed with NP‐40‐free buffer A and then incubated with 40 µL of buffer B (containing 5 mM HEPES, 1.5 mM MgCl₂, 0.2 mM ethylenediaminetetraacetic acid (EDTA), 0.5 mM DTT, 300 mM NaCl, 26% glycerol, 1× PI, 574 μM PMSF, 1 mM NaVO₄, 1 mM NaF, 2.5 mM NaPPi and 1 mM β‐GP to extract nuclear proteins. The samples were lysed mechanically by repeated aspiration through a needle and incubated on ice for 30 min. The samples were then centrifuged at 15000 × *g* for 10 min at 4°C. The supernatant, containing nuclear proteins, was collected.

### 2.8 Western Blot Analysis

Protein concentration was determined spectrophotometrically using the Bradford assay, in which 1 µL of each sample was added to Bradford reagent diluted in a solution containing 80% water and 20% Bio‐Rad protein assay reagent. Thirty micrograms (30 μg) of total protein extracts were separated on a 10% or 12% SDS‐PAGE, transferred to a nitrocellulose membrane, and probed with an antibody for pro‐caspase 7 (sc‐56063, Santa Cruz Biotechnology, Dallas, TX, USA), diluted 1:1000; MFN2 (9482S, Cell Signaling, Danvers, MA, USA) diluted 1:000; p‐DRP1[S616] (4494S, Cell Signaling, Danvers, MA, USA) diluted 1:1000; DRP1 (DLP1,611112, BD biosciences, Franklin Lakes, NJ, USA) diluted 1:1000; Nrf2 (16396‐1‐AP, Proteintech, San Diego, CA, USA) diluted 1:1000; MBP (NE1019, Sigma‐Aldrich, St. Louis, MO, USA) and PLP (sc‐517649, Santa Cruz Biotechnology, Dallas, TX, USA) diluted 1:250 in 5% BSA in 20 mM Tris‐buffered saline, pH 7.6, 0.1% Tween 20 (TBST), and with an antibody for α‐tubulin (T9026, Sigma‐Aldrich, St. Louis, MO, USA) diluted 1:5000 in 5% BSA in TBST. Following primary antibody incubation, membranes were washed and incubated with species‐specific horseradish peroxidase‐conjugated secondary antibodies: anti‐rabbit IgG‐HRP (1706515, Bio‐Rad, Hercules, CA, USA) and anti‐mouse IgG‐HRP (1706516, Bio‐Rad, Hercules, CA, USA), each diluted 1:5000 in 5% BSA in TBST. Signals were developed using the ECL substrate kit (Elabscience, Houston, TX, USA). Images were acquired using the ChemiDoc™ MP Imaging System with Image Lab™ Software (Bio‐Rad, Hercules, CA, USA). Band intensity analysis was analyzed using ImageJ Software (National Institutes of Health, Bethesda, MD, USA). When required, membranes were stripped using a commercial stripping solution (Sigma‐Aldrich, St. Louis, MO, USA) and reprobed to detect proteins with similar electrophoretic mobility due to comparable molecular weights, such as p‐DRP1 and DRP1.

For nuclear and cytosolic protein fractionation, a 1:5 ratio of nuclear to cytosolic protein was maintained; accordingly, 30 μg of cytosolic and 6 μg of nuclear proteins were subjected to electrophoresis.

### 2.9 Gene Expression Analysis

MO3.13 cells were grown to semi‐confluence in 12‐well plates in complete DMEM and subsequently starved for 24 h with 0.2% FBS medium in the absence, in presence of PA 25 μM or in differentiation medium (PMA). Total RNA was extracted from cell cultures using the TRIzol Reagent (Thermo Fisher Scientific, Waltham, MA, USA), following the manufacturer's instructions. For quantitative Real Time‐PCR (qPCR), cDNAs were produced using 1 μg total RNA and the iScript cDNA Synthesis Kit from Bio‐Rad (Bio‐Rad, Hercules, CA, USA) following manufacturer's instruction. Aliquots of cDNAs were then used in qPCRs conducted on CFX96 real‐time system instruments (Bio‐Rad, Hercules, CA, USA) using SensiFAST SYBR No‐ROX (Bio‐Line). Beta 2‐microglobulin mRNA was used for internal normalization. Relative fold variations were calculated using the 2^−ΔΔCt^ method described by Livak and Schmittgen (Livak and Schmittgen 2001). The primers used are listed in Table 1.

**Table 1:** Primers used for qPCR analysis.

Gene	Primer Fwd (5’–3’)	Primer Rev (5’–3’)
*β2M*	CCGTTGGCCTTAGCTGTGCT	TCGGATGGATGAAACCCAGA
*CD36*	GGCTGTGACCGGAACTGTG	AGGTCTCCAACTGGCATTAGAA
*GCLM*	GACAAAACACAGTTGGAACAGC	CAGTCAAATCTGGTGGCATC
*NQO1*	CAGCTCACCGAGAGCCTAGT	TAGAGGTCCGACTCCACCAC
*PPARγ*	TTGCTGTCATTATTCTCAGTGGA	GAGGACTCAGGGTGGTTCAG

### 2.10 Lipid Droplet Evaluation

Cells (1 × 10^4^) were plated on glass and, after 18 h, were cultured in 0.2% FBS and treated with PA 25 µM or in differentiation medium (PMA). Cells were then fixed with 3.7% PFA for 15 min at room temperature, washed with PBS, and stained with BODIPY 493/503 probe (Sigma‐Aldrich, St. Louis, MO, USA) (excitation/emission 493/504 nm) at a concentration of 1 µg/mL for 30 min. Nuclei were stained with DAPI (Sigma‐Aldrich, St. Louis, MO, USA). Images were acquired using a LEICA DMi8 fluorescence microscope equipped with Leica Application Suite LAS X Imaging Software. BODIPY positive LDs pixel areas were analyzed using ImageJ software (National Institutes of Health, Bethesda, MD, USA). Five images per sample were analyzed (Di Lorenzo et al. 2025).

### 2.11 Wound Healing Assays

To assess cell migration *in vitro*, a wound healing assay was performed. Briefly, 23 × 10^4^ cells were seeded in 6‐well plates in complete DMEM. An artificial wound was manually generated with a 200 µL plastic tip, and cultures were photographed with a light microscope (LEICA DMi, Leica Microsystems, Wetzlar, Germany). Then, cells were washed with PBS and incubated with PA 25 µM in complete medium or in differentiation medium (PMA), both supplemented with 0.5 mg/mL mitomycin‐C to inhibit cell proliferation. At zero time point (T_0_) and/or after 24 h (T_24_), the medium was removed, and cells were immediately fixed with 3.7% PFA for 15 min at room temperature. Cells were washed with PBS and then were stained with Coomassie Brilliant Blue for 30 min at room temperature and rinsed with distilled water. To visualize the result of the scratch assay, digital images of the wells were acquired with a Leica DMi1 light microscope (10× objective) and analyzed quantitatively with ImageJ software (National Institutes of Health, Bethesda, MD, USA). Five images were analyzed for each sample. Results were shown as percent wound closure calculated as (area T_0_ – area T_24_)/area T_0_×100.

### 2.12 Cell Cycle Analysis

For cell cycle analysis, 23 × 10^4^ cells were plated in 6‐well plates in complete DMEM. The following day, they were placed in medium with 0.2% FBS for 18 h, except for the growing control, to promote cell synchronization. Then, the medium was replaced with complete DMEM supplemented with PA 25 µM to assess its effect, or with differentiation medium containing PMA as a positive control for cell cycle arrest. Subsequently, the samples were trypsinized, washed with PBS, and centrifuged for 5 min at 200 × *g*. The cell pellets were resuspended in 200 µL of buffer (0.1% Triton X‐100, 0.1 mg/mL RNase A, and 1 mg/mL PI in PBS) for 30 min. The samples were analyzed using a FACSCanto II flow cytometer (BD, Franklin Lakes, NJ, USA). Data analysis was conducted using FlowJo software (v. 10, BD, Franklin Lakes, NJ, USA) with the DEAN‐JET‐FOX model.

### 2.13 Immunofluorescence Microscopy Analysis

MO3.13 cells were grown on coverslips in the presence of 0.2% FBS and PA 25 µM or in differentiation medium (PMA) for 4 days. For MBP staining, the cell medium was removed, and the cells were immediately fixed in 3.7% PFA in PBS with 2% sucrose (pH 7.4), for 5 min at 22°C. After two washes in PBS with 2% sucrose, they were permeabilized for 10 min at 4°C with 0.005% saponin (Sigma‐Aldrich, from quillaja bark, St. Louis, MO, USA) in PBS. After saturating the nonspecific binding sites with 20% FBS in PBS for 1 h at 4°C, the cells were incubated with human anti‐MBP primary polyclonal antibody (1:200, AB980, Millipore Upstate) for 2 h at room temperature. The cells were washed and incubated with the secondary antibody anti‐donkey conjugated with Alexa Fluor 488 (1:300 dilution, Invitrogen, Thermo Fisher Scientific, Waltham, MA, USA) for 1 h at room temperature. Control samples were incubated with secondary antibody only (Ab II). Nuclei were stained with DAPI (1 µg/mL; Sigma‐Aldrich, St. Louis, MO, USA). Coverslips were then washed with PBS followed by distilled water and mounted on slides for microscopic examination. The images were acquired using a Leica DMi8 microscope with a 20X objective and analyzed using ImageJ software (National Institutes of Health, Bethesda, MD, USA) according to the protocol as previously described.

### 2.14 Animals

All animal experiments and animal handling and care were in accordance with the ARRIVE guidelines and the Guide for the Care and Use of Laboratory Animals (EU Directive 2010/63/EU), and the experimental protocol was approved by the Animal Care and Use Committee of “Federico II” University of Naples, Italy, and Ministry of Health, Italy. Female Wistar rats (14‐days timed pregnant) were obtained from Charles River Laboratories (Italy) and maintained at a constant temperature (22 + 1°C) on a 12 h light/dark cycle (lights on at 7 AM) with food and water ad libitum. Welfare‐related assessments were carried out serially during the study. The pregnant dams were allowed to deliver their pups naturally; 7‐10 days postpartum littermates were used for the preparation of hippocampal and cerebellar organotypic explants. All efforts were made to minimize animal suffering and to reduce the number of animals used. Both male and female animals were used for organotypic explant preparation.

### 2.15 Hippocampal and Cerebellar Organotypic Explants

Organotypic explants were prepared as previously described (Boscia et al. 2008). Postnatal P10 day‐old Wistar rat pups (Charles River Laboratories, Calco, Italy) were killed by cervical dislocation and 400 μm‐thick parasagittal slices were obtained from dissected hippocampi or cerebellum, respectively, using a McIlwain tissue chopper (Campden Instruments, Leicester, UK) and placed into ice‐cold Hank's balanced salt solution (HBSS, Gibco, Thermo Fisher Scientific, Milan, Italy) supplemented with 5 mg/mL glucose and 1.5% (v/v) fungizone. Cultures were then transferred to a humidified semiporous membrane, 30 mm Millicell tissue culture plate inserts of 0.4 mm pore size (Millipore, Milan, Italy) in 6‐well tissue culture plates (5 slices per membrane). Each well contained 1.2 mL of tissue culture medium consisting of 50% minimal essential medium (MEM, Gibco, Thermo Fisher Scientific, Milan, Italy), 25% HBSS, 25% heat inactivated horse serum (HS, Gibco, Thermo Fisher Scientific, Italy), 6.5 mg/ml glucose, 1 mM glutamine, and 1.5% fungizone (Thermo Fisher Scientific, Italy) (normal medium, NM). Cultures were maintained at a 37°C and 5% CO^2^‐conditioned atmosphere.

### 2.16 Assessment of Cell Death in Hippocampal Explants and Image Analysis of Neurodegeneration

Hippocampal explants were exposed to a combined application of 10 µg/mL lipopolysaccharide (LPS, Merck Life Science S.r.l., Milan, Italy) plus 100 ng/ml interferon‐γ (IFN‐γ, Thermo Fisher Scientific, Milan, Italy) for 4 days, as previously described (Cammarota et al. 2023; Cammarota and Boscia 2023).

Hippocampal slice cultures represent the gold standard *ex‐vivo* 3D model for monitoring neurodegeneration in a variety of insults, due to their well‐characterized region vulnerability (Boscia et al. 2006). Hippocampal explant exposure to LPS + IFN‐γ triggers the release of massive proinflammatory and cytotoxic factors, and promotes demyelination and inflammatory neurodegeneration (Cammarota et al. 2023; Cammarota and Boscia 2023; Papageorgiou et al. 2016). Furthermore, the selective neurodegeneration occurring in the CA1 hippocampal region following LPS + IFN‐γ exposure can be monitored by PI uptake, providing a platform to investigate the effects of compounds against the neuroinflammatory damage (Cammarota and Boscia 2023).

Experiments were performed on cultures kept *in vitro* for 10 days (10 DIV). Appropriate concentrations of PA 25 µM, or vehicle (BSA 5%) were added to the medium at the beginning of the insult and were kept in the culture medium during the entire duration of the experiment. Control culture explants were kept in the absence or in the presence of testing compounds. Cell injury was assessed in hippocampal explants by live incorporation of a marker of compromised membrane integrity, PI 5 µg/mL (Thermo Fisher Scientific, Milan, Italy), that emits a bright red fluorescence when exposed to blue‐green light. For densitometric measurements, the digital pictures were analyzed with the Image Pro‐Plus software (Media Cybernetics), after freehand outlining of the CA neuronal layer, as previously described (Cammarota et al. 2023).

### 2.17 Confocal Microscopy in Cerebellar Explants and Image Analysis of Myelination and Remyelination

Myelination and remyelination studies were performed in organotypic cerebellar slice cultures, as previously described (de Rosa et al. 2019). Cerebellar explant cultures provide a model to investigate developmental myelination, while explant exposure to lysophosphatidylcholine (LPC) represents a widely used and well‐established *ex‐vivo* model of demyelination and subsequent remyelination. This model is the most effective *ex‐vivo* system for studying the interaction between oligodendrocytes and axons and the effect of compounds on remyelination rate within a preserved 3D architecture (de Rosa et al. 2019; Doussau et al. 2017; Zhang et al. 2011).

For myelination studies, slices cultures were kept in culture for 4 days in vitro (DIV) to allow tissue recovery from the dissection and then cultured in the presence or in the absence of PA 25 μM from 5 DIV until 10 DIV. Fresh medium and PA was replaced every 2 days and all slices were maintained for 10 DIV, when they were collected for further analysis.

For remyelination studies, 12 DIV cerebellar organotypic slices were demyelinated with 0.5 mg/mL lysophosphatidylcholine (LPC, Sigma‐Aldrich, Milan, Italy) for 15–17 h. Then, slices were washed in normal medium for 5 min and treated at 1 day postlysolecithin (dpl) with PA 25 μM until 8 dpl.

Confocal immunofluorescence procedures in organotypic explants were performed as previously described (de Rosa et al. 2019; Casamassa et al. 2016). Slices were fixed in 4% w/v paraformaldehyde in phosphate buffer (PB), blocked with 3% BSA (Merck Life Science S.r.l., Milan, Italy, Cat# 9036‐19‐5) for 1 h and then incubated with the following primary antibodies: rabbit polyclonal anti‐neurofilament‐200 (anti‐NF200; 1:500, Merck Life Science S.r.l., Milan, Italy,), mouse monoclonal anti‐MBP (1:1000, Merck Life Science S.r.l., Milan, Italy), After 48 h at 4°C, slices were incubated with the corresponding secondary antibodies for 2 h at room temperature. Finally, they were mounted on glass slides and imaged with Zeiss LSM700 laser (Carl Zeiss, Jena, Germany) scanning confocal microscope.

Confocal microscopy was used to obtain stacks of photographs of MBP/NF200 immunolabelling at 2 μm intervals in representative myelinated regions of cerebellar slices at 40× magnification and a resolution of 1024 × 1024, between a depth of 5–20 μm from the upper surface. Four independent slices from three different animals were analyzed per condition. Five images of randomly chosen areas of each slice were acquired with identical fluorescence intensity. Maximum intensity projection (MIP) images were created for each stack by using ZEN imaging software. Then, for each MIP image quantification of colocalization between MBP and NF200 immunostaining (myelination index) was assessed by using the ‘co‐localization highlighter's plug‐in for the ImageJ software (NIH, Bethesda, MD, USA) (de Rosa et al. 2019). Before colocalization analysis, images were first thresholded to identify the positive signal. Subsequently, the number of pixels positive for MBP/NF200 was measured per microscope field. This value, expressed as percentage of co‐localization, represents the extent of MBP/NF200 co‐expression.

### 2.18 Statistical Analysis

Statistical analysis was conducted using T‐test Students for two groups comparison. For multiple comparisons of more than two groups, one‐way or two‐way ANOVA followed by Bonferroni's post hoc test was used (GraphPad Prism 6.0; GraphPad Software Inc., La Jolla, CA, USA). *p* < 0.05 were considered as statistically significant (*p < 0.05, **p < 0 .01, ***p < 0.001, ****p < 0.0001). All data are reported as mean ± SEM from at least three independent experiments performed in triplicate.

## 3 Results

### 3.1 Dose‐Dependent Impact of Palmitic Acid on Oligodendrocyte Viability and Mitochondrial Metabolic Activity

**Figure 1:**
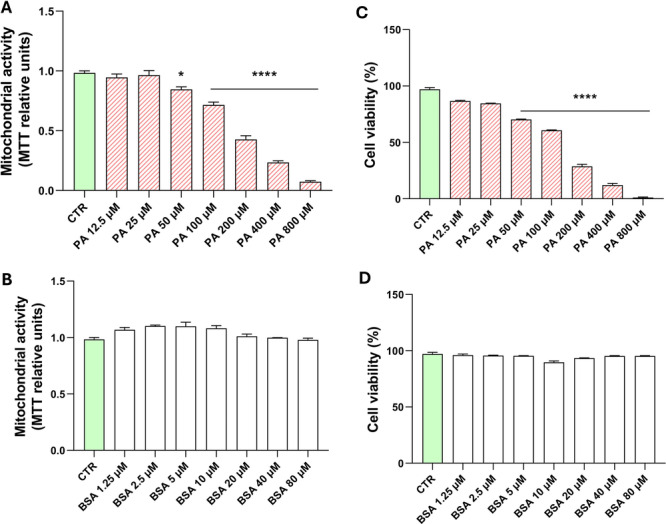
Effect of PA on gross mitochondrial activity and cell viability. MO3.13 were incubated for 24 h in absence (CTR) or in the presence of increasing doses of PA and BSA (vehicle). The molar ratio between PA and BSA is 10:1. Gross mitochondrial activity was measured by (A, B) MTT assay, while cell viability was measured by (C, D) trypan blue staining. The histograms show the means ± SEM of three independent experiments in triplicate. Statistical analysis was obtained by Ordinary one‐way ANOVA with Bonferroni's post hoc test correction. *p < 0.05 versus CTR, ****p < 0.0001 versus CTR.

The effect of different concentrations of PA on gross mitochondrial activity and cell viability in MO3.13 cells was assessed using the MTT assay (Figure 1A, B) and the trypan blue exclusion assay (Figure 1C, D), respectively. The concentrations of palmitic acid were selected based on their physiological relevance to plasma levels observed in humans (Yi et al. 2007), as well as their common use in *in vitro* studies (Wang et al. 2014). A significant reduction in both mitochondrial activity and cell viability was observed after 24 h of treatment, starting at 50 µM. The MTT assay revealed a dose‐dependent decrease in mitochondrial activity, indicating that high concentrations of PA impair cellular metabolic function. Consistently, the trypan blue assay confirmed a dose‐dependent increase in cell death: PA at 12.5 and 25 µM showed no cytotoxic effects, while 50 and 100 µM induced mild cytotoxicity, and higher concentrations caused moderate to strong cytotoxicity. For both assays, no significant effects were observed with the corresponding concentrations of the vehicle (BSA) (Figure 1B, D).

### 3.2 High Dose of Palmitic Acid Induces Apoptotic Death

Given the reduction in cell viability and gross mitochondrial activity following PA treatments, we evaluated whether apoptosis was the underlying mechanism. For the experiments, PA 25 µM, the higher non‐cytotoxic dose, and PA 100 µM which maintained cell viability above 50%, were selected for the analysis.

**Figure 2:**
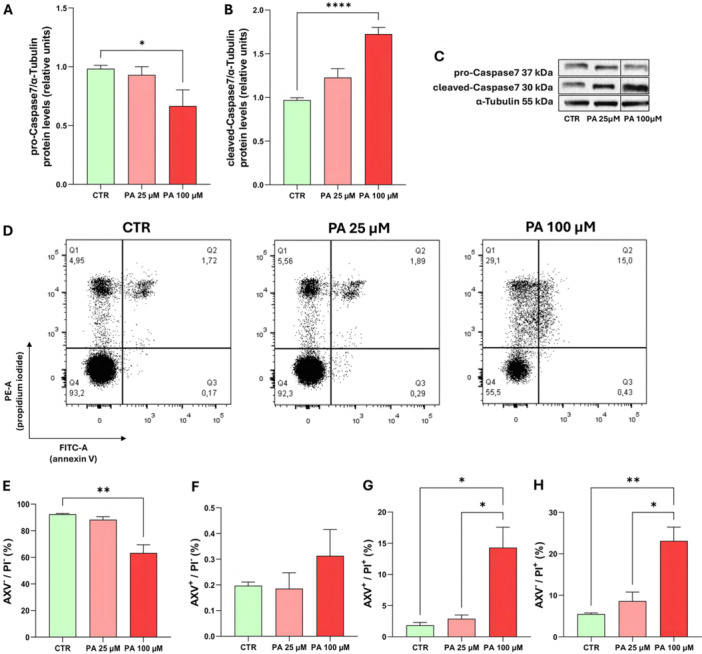
High doses of PA induce apoptosis. MO3.13 cells were cultured for 24 h in absence (CTR) or in presence of PA (25, 100 µM). Cell death was assessed by western blot analysis (caspase7) and using markers of apoptosis and necrosis (AXV/PI). (A–C) Representative Western blot image and histograms resulting from densitometric analysis of pro‐caspase 7 and cleaved‐caspase 7 protein bands normalized on α‐tubulin. The omitted lanes do not contain data pertinent to the study's conclusions. (D) Representative images of AXV/PI staining. (E) percentage of AXV^‐^/PI^‐^ cells, (F) percentage of AXV^+^/PI^‐^ cells, (G) percentage of AXV^+^/PI^+^ cells, (H) percentage of AXV^‐^/PI^+^ cells. The histograms show the means ± SEM of three independent experiments in triplicate. Statistical analysis was obtained by Ordinary one‐way ANOVA with Bonferroni's post hoc test correction. *p < 0.05, **p < 0.01, ***p < 0.001, ****p < 0.0001.

Western blot analyses revealed that at PA 25 µM no significant changes in both inactive pro‐caspase 7 and active cleaved caspase 7 levels were observed. However, at PA 100 µM, cleaved caspase‐7 levels increased while the inactive form decreased (Figure 2A–C).

These data show that PA 25 µM does not induce apoptosis, whereas PA 100 µM triggers caspase‐mediated apoptotic cell death likely via the intrinsic pathway.

To confirm these findings, cell death was assessed by flow cytometry using two dyes, annexin V (AXV) and propidium iodide (PI). Flow cytometric analysis of AXV/PI staining allows the identification of different types of cell death: early apoptosis (AXV⁺/PI⁻), late apoptosis (AXV⁺/PI⁺), and necrosis (AXV⁻/PI⁺) (Figure 2D).

At PA 100 µM, the % of viable (AXV^‐^/P^‐^) cells (Figure 2E) significantly decreased, while no significant differences were observed in early apoptotic (AXV^+^/PI^‐^) cells (Figure 2F). In addition, it was observed that the % of late apoptotic (AXV^+^/PI^+^) (Figure 2G) and necrotic (AXV^‐^/PI^+^) (Figure 2H) cell populations increased significantly at PA 100 µM compared to control. The increased number of AXV^+^/PI^+^ cells after treatment with PA 100 µM suggests progression from early to late apoptosis and/or necrosis. At PA 25 µM, apoptosis/necrosis levels remained as low as those observed in control conditions.

Overall, results from AXV/PI staining and caspase‐7 cleavage assays demonstrate that PA 100 µM is a potent inducer of programmed cell death in OLs.

### 3.3 Palmitic Acid Exerts Biphasic Control of Mitochondrial Homeostasis

Mitochondria play a crucial role in OL maturation, differentiation, and survival (Damiano et al. 2021). FAs elicit mitochondrial bioenergetic reprogramming, potentially affecting both their functional efficiency and dynamics (Ramachandra et al. 2018; Cecatto et al. 2020). Therefore, mitochondrial morphology, using the MitoTracker Red CMXRos fluorescent probe, and the expression levels of proteins involved in mitochondrial dynamics in MO3.13 cells were evaluated.

**Figure 3:**
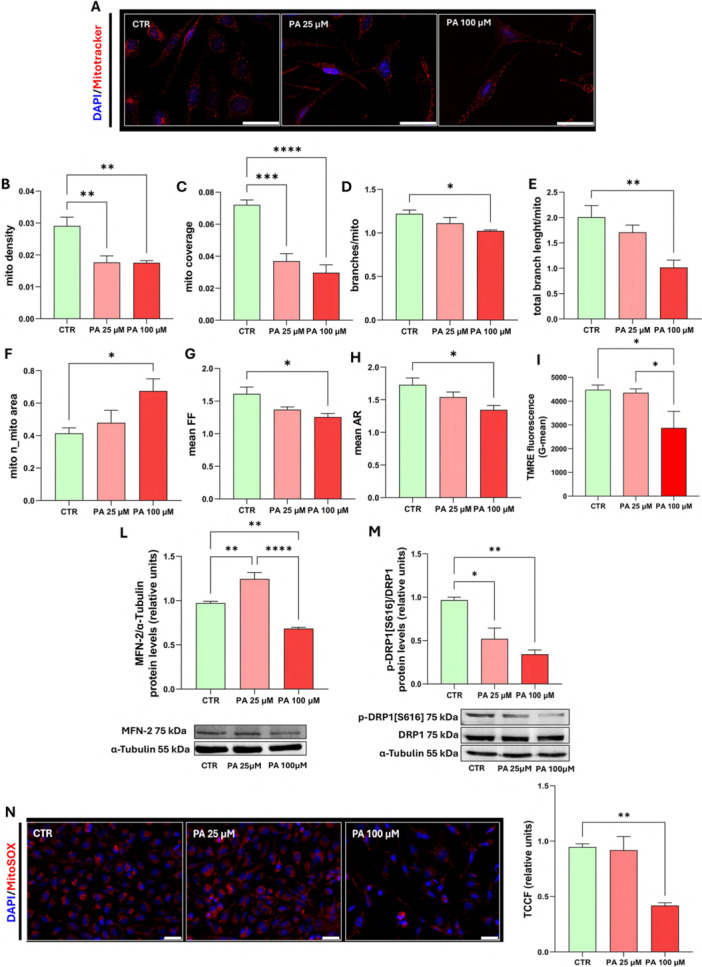
*Dose‐dependent effects of PA on mitochondrial dynamics, morphology*, ΔΨm *and mtROS*. MO3.13 cells were treated for 24 h in absence (CTR) or in presence of PA (25, 100 µM). To evaluate mitochondrial morphology, mitochondria were labeled with the MitoTracker Red CMXRos probe. (A) Fluorescence images (scale bar 50 µm) of the Mitotracker staining, (B) Analysis of mitochondrial density, (C) mitochondrial coverage, (D, E) mitochondrial network integrity, (F) mitochondrial number/mitochondrial area ratio, (G) form factor (FF) and (H) Aspect ratio (AR). The histograms show the means ± SEM of five independent experiments in triplicate. (I) ΔΨm was assessed using TMRE by flow cytometry. The histograms show the means ± SEM of three independent experiments in triplicate. (L) Representative Western blot image and histograms resulting from densitometric analysis of MFN2 protein bands normalized on α‐tubulin. The histograms show the means ± SEM of four independent experiments in triplicate. (M) Representative Western blot image and histograms resulting from densitometric analysis of p‐DRP1[Ser616] protein bands normalized on α‐tubulin and total DRP1. The histograms show the means ± SEM of three independent experiments in triplicate. (N) mtROS were labeled with MitoSOX fluorescent probe. Representative fluorescence images (scale bar 50 µm) for MitoSOX staining and quantitative analysis of mtROS are shown. The histograms show the means ± SEM of three independent experiments in triplicate. Statistical analysis was obtained by Ordinary one‐way ANOVA with Bonferroni's post hoc test correction. *p < 0.05, **p < 0.01, ***p < 0.001, ****p < 0.0001.

Quantitative analysis of fluorescence images (Figure 3A) allowed us to evaluate key parameters describing mitochondrial network structure and integrity following PA treatment. Mitochondrial density refers to the number of mitochondria per cytoplasmic area (Figure 3B) while coverage (Figure 3C) represents the proportion of the cytoplasm area occupied by mitochondria. Both parameters significantly decreased at PA 25 and 100 µM, indicating a reduction in the mitochondrial network extension.

At the higher concentration PA (100 µM) treatment also significantly reduced the number of mitochondrial branches (Figure 3D) and total branch length for each mitochondrion (Figure 3E). Furthermore, PA 100 µM induced a significant increase in the ratio of the mitochondrial number over total mitochondrial area (Figure 3F).

To further assess mitochondrial morphology and dynamics, we analyzed the form factor (FF) (Figure 3G) which measures mitochondrial complexity. A higher FF indicates a more interconnected and branched network. A significant FF reduction was observed only at PA 100 µM. Similarly, the aspect ratio (AR), a measure of mitochondrial elongation, calculated as the ratio between the major and minor axes significantly decreased at PA 100 µM suggesting a shift toward mitochondrial fragmentation. Under control conditions and in the presence of PA 25 µM (Figure 3H), mitochondria predominantly exhibited a tubular shape, characteristic of a healthy mitochondrial network. TMRE analysis (Figure 3I) revealed distinct effects of PA concentrations on mitochondrial membrane potential (ΔΨm). In line with previous data, treatment with PA 100 µM led to a marked decrease in ΔΨm, which is typical of apoptotic cells. Conversely, PA 25 µM did not cause significant changes in ΔΨm compared to the control.

Treatment with PA 100 µM was able to reduce ΔΨm, mitochondrial size and induced a more rounded‐shape, impacting on overall integrity of the mitochondrial network, a phenomenon commonly associated with altered mitochondrial dynamics (Liesa and Shirihai 2013).

Indeed, mitofusin‐2 (MFN2, Figure 3L), a protein involved in mitochondrial fusion, and the active phosphorylated form of Dynamic‐related Protein 1 (p‐DRP1[Ser616], Figure 3M), a protein involved in mitochondrial fission, are both reduced in the presence of high doses of PA, confirming mitochondrial dysfunction at PA 100 µM (Yapa et al. 2021; Wang et al. 2009; Vezza et al. 2022). Data obtained with PA 25 µM on the same proteins show a statistically significant increase in MFN2 and a decrease in p‐DRP1[Ser616] over total DRP1 compared to the control, suggesting a shift toward mitochondrial fusion.

These data evidenced that high‐dose PA disrupts mitochondrial function, ultimately leading to apoptosis, as shown above.

Since mitochondria are the main source of ROS, to highlight an association between alterations in mitochondrial dynamics and mitochondrial function, the impact of PA on ROS was assessed.

Mitochondrial ROS (mtROS) production was measured by determining the fluorescence intensity of the MitoSOX probe (Figure 3N). As observed in fluorescence images and corresponding histograms, treatment with PA 25 µM did not result in a significant variation in ROS levels compared to control. Interestingly, at PA 100 µM, the MitoSOX signal strongly decreased suggesting that mitochondrial dysfunction has progressed to a point where damaged mitochondria lose their ability to generate ROS, likely due to severe structural and functional deterioration.

### 3.4 Low Dose of Palmitic Acid Reduces Total ROS Levels by the Activation of NRF2

**Figure 4:**
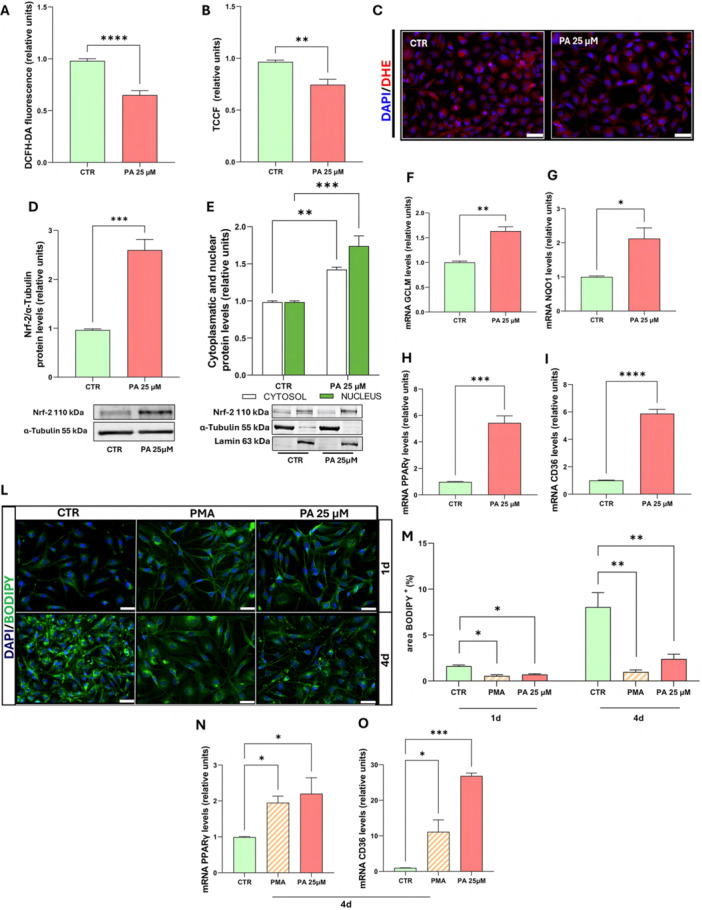
Low dose of PA induce an Nrf2‐mediated antioxidant response and induces lipid mobilization. MO3.13 cells were cultured for 24 h in the absence (CTR) or in the presence of PA 25 µM. (A) Quantitative analysis of intracellular ROS levels measured with the DCFH‐DA fluorescent probe. The histograms show the means ± SEM of eight independent experiments in triplicate. (B, C) Quantitative analysis of ROS levels measured with the DHE fluorescent probe and representative fluorescence images (scale bar 50 µm) for the DHE staining. The histograms show the means ± SEM of five independent experiments in triplicate. (D) Representative Western blot image and histograms resulting from densitometric analysis of total Nrf2 protein bands normalized on α‐tubulin. The histograms show the means ± SEM of four independent experiments in triplicate. (E) Representative Western blot image and histograms resulting from densitometric analysis of nuclear and cytosolic Nrf‐2 protein bands normalized on lamin A/C. The histograms show the means ± SEM of three independent experiments in triplicate. (F‐I) qPCR analysis of Nrf2 downstream targets involved in the oxidative stress response. The histograms show the means ± SEM of three independent experiments in triplicate. (L, M) MO3.13 were incubated for 1 or 4 days (1 or 4 d) in absence (CTR), in presence of PA 25 µM or in differentiation medium (PMA). Lipid accumulation in OLs were measured after 1 and 4 days using BODIPY staining. Representative images (scale bar 50 µm) for BODIPY 493/503 fluorescence staining and related lipid droplet positive area (LD^+^) analysis. The histograms show the means ± SEM of seven independent experiments in triplicate. (N, O) qPCR analysis of genes involved in lipid metabolism after 4 days of treatment. The histograms show the means ± SEM of three independent experiments in triplicate. Statistical analysis was obtained by Ordinary two‐way ANOVA with Bonferroni's post hoc test correction or *T*‐test Students. *p < 0.05, **p < 0.01, ***p < 0.001, ****p < 0.0001.

Subsequently, the impact of non‐cytotoxic doses of PA on oligodendrocyte function was investigated. ROS are not only markers of oxidative stress but also play a key role as signaling molecules in cellular processes, including differentiation and myelination (Accetta et al. 2016). To assess whether low‐dose PA influences ROS levels, we measured intracellular ROS using the specific probe DCFH‐DA (Figure 4A). After 24 h treatment with PA 25 µM, a significant reduction in total ROS was observed. This finding was further confirmed by fluorescence microscopy using the superoxide‐specific probe DHE (Figure 4B, C). This reduction may result from a cellular adaptive response mediated by Nrf2. Indeed, Western blot analysis revealed a marked increase in total Nrf2 levels and its nuclear translocation (Figure 4D, E) following 24 h PA stimulation. Furthermore, qPCR experiments confirmed Nrf2 activation, showing upregulation of key oxidative stress response genes downstream of Nrf2 (Figure 4F, G). Specifically, we observed an increased expression of glutamate‐cysteine ligase modifier subunit (GCLM), the rate‐limiting enzyme in glutathione (GSH) synthesis, NAD(P)H quinone oxidoreductase 1 (NQO1), an enzyme crucial for quinone detoxification and oxidative stress mitigation. Additionally, since Nrf2 is linked to peroxisome proliferator‐activated receptor gamma (PPARγ), a transcription factor involved in lipid and glucose metabolism, inflammation modulation, and cell differentiation (De Nuccio et al. 2020), we tested the mRNA levels of PPARγ and the cluster of differentiation 36 (CD36) (Figure 4H, I), a receptor responsible for cellular FA transport, under the same experimental conditions. The results demonstrated that the expression of both genes was upregulated. These data suggest that OLs can actively respond to low doses of PA exposure through an adaptive Nrf2‐mediated mechanism.

### 3.5 Low Dose of Palmitic Acid Reshapes Lipid Metabolism During Oligodendrocyte Maturation

Beyond its role in oxidative stress regulation, Nrf2 activation is also linked to metabolic adaptations, including lipid metabolism and differentiation. The effects of palmitic acid (PA) on Nrf2, CD36 and PPARγ, which is involved in OLs differentiation (Bernardo et al. 2021, 2009), suggest a role for PA in these two processes. To examine this hypothesis, lipid accumulation was analyzed using BODIPY staining at early (1 day) and late (4 days) stages of treatment. Additionally, treatment with Phorbol‐12‐Myristate‐13‐Acetate (PMA) in FBS‐free medium was included as a known positive control for the differentiation of this cell line. Fluorescence imaging and quantification (Figure 4L, M) revealed that under control conditions, lipid droplets (LDs) accumulation increased over time, with a higher LDs content observed at 4 days compared to 1 day, likely reflecting the storage of unused FAs. In contrast, treatment with PA 25 µM significantly reduced LDs content after 24 h, and this reduction was maintained at 4 days. Similarly, PMA treatment also led to a marked decrease in LDs accumulation compared to controls, suggesting enhanced mobilization and utilization of lipids in cells undergoing differentiation.

Consistently, gene expression analysis showed that both PPARγ and CD36 (Figure 4N, O) remained markedly upregulated even after 4 days of PA exposure. An increase in PPARγ and CD36 expression was also observed in cells treated with PMA. These findings suggest that PA promotes a metabolic shift characterized by reduced lipid storage and increased expression of genes involved in lipid uptake, supporting the hypothesis that PA facilitates metabolic adaptations linked to oligodendrocyte maturation.

### 3.6 Low Dose of Palmitic Acid Modulates Early Oligodendrocyte Maturation

**Figure 5:**
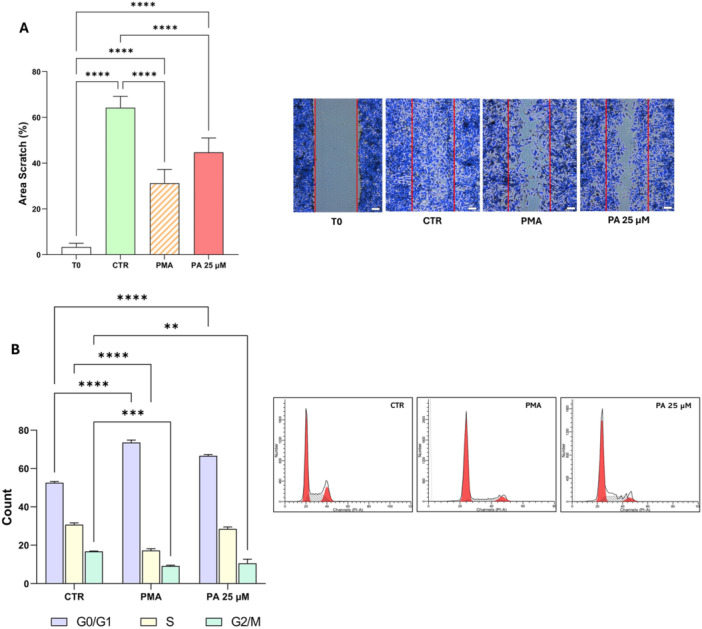
Low dose of PA inhibits migration and cell cycle progression. MO3.13 cells were incubated for 24 h either in the absence of treatment (CTR), or in the presence of 25 µM palmitic acid (PA) or differentiation medium (PMA). The inhibition of early oligodendrocyte maturation processes after treatments was assessed through scratch assay and cell cycle analysis. (A) Quantitative analysis of the covered scratch area (expressed as percentage of pixels) and representative image (scale bar 50 µm) of the scratch assay. The histograms show the means ± SEM of five independent experiments in triplicate. (B) Quantitative analysis of cells in the G0/G1, S and G2/M phases and representative image of the cell cycle. The histograms show the means ± SEM of three independent experiments. Statistical analysis was obtained by Ordinary one‐way or two‐way ANOVA with Bonferroni's post hoc test correction. *p < 0.05, **p < 0.01, ***p < 0.001, ****p < 0.0001.

OPCs migration is a key early event in the maturation process of OPCs, ensuring proper positioning before their differentiation into OLs. Our data indicate that PA 25 µM significantly reduces cell migration as observed in the scratch assay after 24 h (Figure 5A). A similar reduction was detected in PMA‐treated cells, compared to the control condition. Furthermore, cell cycle analysis (Figure 5B) revealed a G0/G1 phase arrest, leading to a lower proportion of cells progressing to the G2/M phase following PA 25 µM or PMA treatment compared to the control. These findings suggest that PA modulates early OL maturation by blocking migration and inducing cell cycle arrest, suggesting a shift toward differentiation.

### 3.7 Low Dose of Palmitic Acid Drives Oligodendrocyte Differentiation and Stage‐Dependent Mitochondrial Remodeling

**Figure 6:**
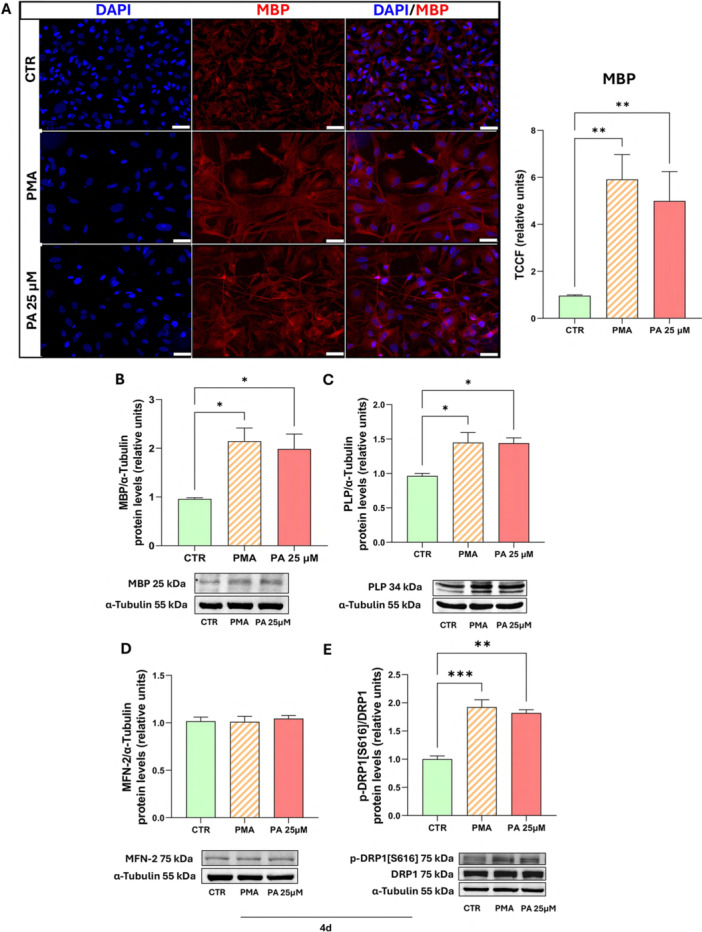
Low dose of PA promotes oligodendrocyte differentiation and modulates mitochondrial dynamics. MO3.13 were incubated for 4 days (4 d) in absence (CTR), in presence of PA 25 µM or in differentiation medium (PMA). Induction of differentiation in MO3.13 after treatment with PA 25 µM or PMA for 4 d was assessed through fluorescence microscopy and Western blot analysis. (A) Representative fluorescence image (scale bar 50 µm) and quantitative analysis of MBP protein expression levels. (B, C) Representative western blot images, densitometric analysis and relative histograms of MBP and PLP normalized with α‐tubulin. (D, E) Representative Western blot image and histograms resulting from densitometric analysis of MFN2 protein bands normalized on α‐tubulin and p‐DRP1[Ser616] protein bands normalized on α‐tubulin and total DRP1. The histograms show the means ± SEM of three independent experiments in triplicate. Statistical analysis was obtained by Ordinary one‐way ANOVA with Bonferroni's post hoc test correction. *p < 0.05, **p < 0.01, ***p < 0.001.

We further evaluated the potential role of PA in differentiation. The expression of myelin protein (MBP) and proteolipid protein (PLP) is a hallmark of differentiated OLs. Fluorescence microscopy images (Figure 6A) reveal that PA 25 µM, like PMA, significantly increases MBP expression levels. Western blot analysis further confirmed these findings, demonstrating increased MBP (Figure 6B) and PLP (Figure 6C) protein levels following PA 25 µM treatment, comparable to the effect observed with PMA. These results confirm the role of low‐dose PA in promoting MO3.13 differentiation.

The involvement of PA 25 µM in oligodendrocyte maturation prompted an investigation into its effects on mitochondrial dynamics in differentiated cells.

It is known that mitochondrial dynamics in mature OLs shift toward fission (Yazdankhah et al. 2021). In line with this, our data show that treatment with PA 25 µM does not alter MFN2 levels. However, after 4 days, it induces an increase in p‐DRP1[Ser616] protein levels, like the PMA‐positive control, when compared to undifferentiated cells (Figure 6D‐E).

These findings further support the role of PA in MO3.13 differentiation, promoting a shift in mitochondrial dynamics toward fission.

### 3.8 Low Dose of Palmitic Acid Exert Protective Effects on Hippocampal Explants Exposed to a Neuroinflammatory Insult

**Figure 7:**
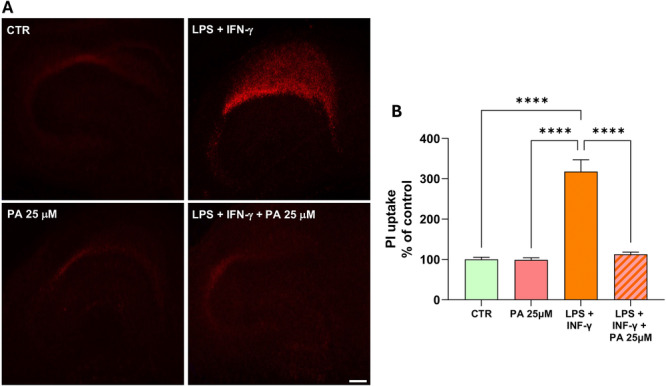
Effects of low dose PA treatment against LPS + IFN‐γ‐induced inflammatory damage in hippocampal explant cultures. (A) PI fluorescence staining patterns observed in representative hippocampal organotypic slices under control conditions (a) or in the presence of PA 25 μM (c); and after 96 h of LPS + IFN‐γ exposure in the absence (b) or in the presence of PA 25 μM (d). Scale bars in a‐d: 500 μm. (B) Quantification of cell damage in the CA1 subfield evaluated by densitometric analysis of PI fluorescence and normalized to that recorded in the CA1 subregion of untreated hippocampal slices. Data are normalized as a percentage of control. The values represent the means ± SEM of three independent experiments (*n* = 3 animals, 3–4 slices per group). Level of significance was determined by using one‐way ANOVA with Bonferroni post hoc analysis. ****p < 0.0001.

Then, to validate the non‐toxic PA dose (25uM) in a more tissue‐like CNS context and determine whether they are preserved under pathological conditions, we investigated the effects of PA on inflammation‐mediated neurodegeneration. To this aim 10 days in vitro (DIV) organotypic explants were exposed to a combined application of LPS and recombinant IFN‐γ, in the absence or in the presence of PA 25 μM. When hippocampal explants were exposed to LPS + IFN‐γ, cell death selectively occurred after 4 days in the CA1 pyramidal cell layer. Densitometric analysis of PI fluorescence showed a significant increase in PI uptake in the CA1 pyramidal layer after LPS + IFN‐γ exposure, which was markedly reduced in slices co‐treated with PA 25 μM, indicating decreased cell death, and supporting the neuroprotective effect of PA. The presence of PA 25 µM in the incubation media throughout the experiment did not significantly affect the viability of organotypic cultures (Figure 7A, B).

### 3.9 Low Dose of Palmitic Acid Enhances Developmental Myelination and Accelerates Remyelination in Cerebellar Organotypic Slices

**Figure 8:**
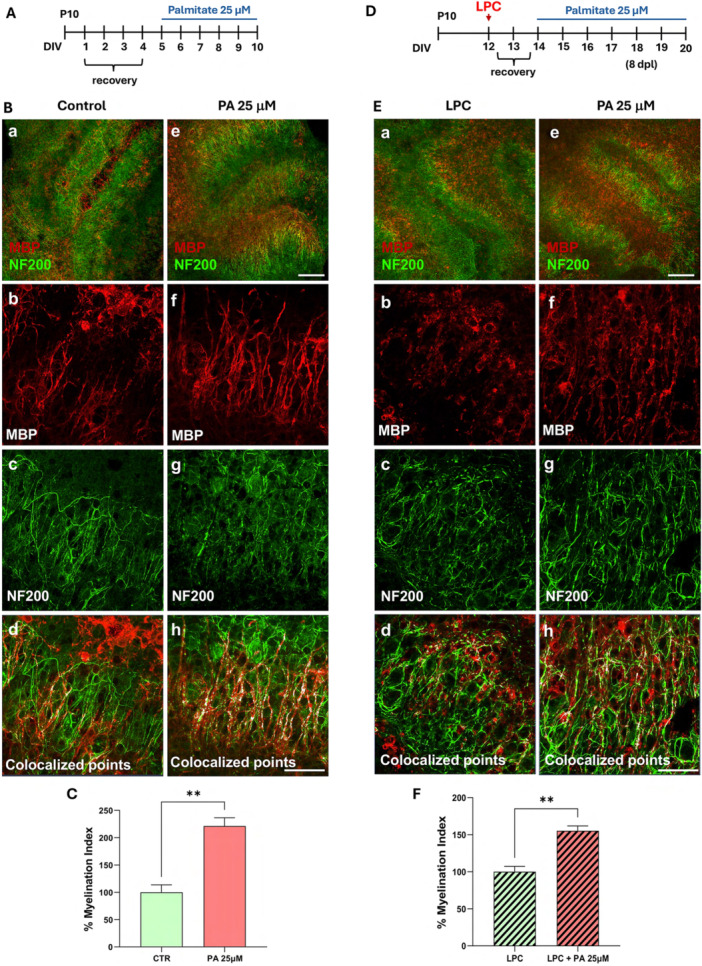
Effect of low dose PA treatment on axonal myelination and remyelination in cerebellar explant cultures. (A) Schematic timeline illustrating PA exposure protocol during developmental myelination in cerebellar organotypic slices. (B) Representative maximum intensity projection of z‐stack confocal images displaying MBP (red) and NF200 (green) immunoreactivities in 10 DIV cerebellar organotypic slices cultured in the absence (b–d) or in the presence of PA 25 μM (f–h). Panels d and h display colocalized points (white). Panels a and e show representative low magnification images of 10 DIV cerebellar slices cultured in the absence (a) or in the presence of PA 25 μM (e). Scale bars in a and e: 200 μm; in b–d and f–h: 50 μm. (C) Analysis of the myelination index in 10 DIV cerebellar slices cultured in the absence or in the presence of PA 25 μM. (D) Schematic timeline illustrating PA exposure protocol after LPC‐induced demyelination in cerebellar organotypic explants. (E) Representative maximum intensity projection of z‐stack confocal images displaying MBP (red) and NF200 (green) immunoreactivities in cerebellar slices at 8 days after LPC exposure (dpl) in the absence (b‐d) or in the presence PA 25 μM (f–h). Panels d and h display colocalized points (white). Panels a and e show representative low magnification images of 8 dpl cerebellar slices cultured in the absence (a) or in the presence of PA 25 μM (e). Scale bars in a and e: 200 μm; in b–d and f–h: 50 μm. (F) Analysis of the myelination index in 20 DIV cerebellar slices cultured after 8dpl in the absence or in the presence of PA 25 μM. The values represent the means ± SEM (*n* = 3 animals, 3–4 slices per group). Statistical analysis was obtained by T‐test Students. **p < 0.01.

To explore whether the non‐toxic PA dose (25 uM) may stimulate axonal myelination, organotypic cerebellar slices were incubated in the presence or in the absence of PA 25 μM during 10 DIV (Figure 8A). Confocal immunofluorescence analysis for MBP and the axonal marker NF200 showed an increased axonal myelination in PA‐treated slices, as revealed by the significant upregulation of the myelination index (colocalization of MBP and axonal neurofilament NF200 staining), compared to control slices (Figure 8B, C).

Next, to investigate the effect of PA treatment on myelin repair we evaluated its effects on axonal remyelination after lysophosphatidylcholine (lysolecithin, LPC) exposure in cerebellar explants. To this aim, cerebellar explants were demyelinated at 12 DIV with LPC and treated at 1 day postlysolecithin (dpl) until 8 dpl with PA 25 μM or vehicle controls (Figure 8D). PA exposure significantly increased remyelination at 8 dpl if compared to LPC‐treated slices, as measured by the myelination index (Figure 8E, F). These data demonstrate that PA 25 μM exposure accelerated remyelination *in vitro*.

## 4 Discussion

This study highlights the biphasic, dose‐dependent effects of PA on OPCs, using the MO3.13 cell line, revealing beneficial effects at low concentrations and toxic outcomes at higher doses. Furthermore, we show that non‐toxic low dose PA exerts protective actions against the neuroinflammatory damage in hippocampal 3D explant cultures and support pro‐myelinating actions in cerebellar *ex‐vivo* explants, both during developmental myelination and in a model of myelin damage and repair.

Our data indicate that while high doses of PA (100 µM) induce mitochondrial dysfunction and apoptosis, a low, non‐cytotoxic PA dose (25 µM) promotes antioxidant responses and lipid mobilization, thereby driving OPC differentiation into more mature OLs. This dual effect underlines the complex biological role of PA on oligodendrocytes and its potential relevance in demyelinating diseases such as MS.

An excess of SFAs alters homeostatic mechanisms, causes oxidative stress, and triggers inflammatory responses (Sørensen et al. 2010). Indeed, it is well known that astrocytes exposed to PA also exhibit inflammatory phenotypes, highlighting the contribution of glial cells to neuroinflammatory pathways (Wong et al. 2014). We found that high concentrations of PA promote oligodendrocyte death, as demonstrated by enhanced AXV/PI staining, and caspase 7 activation, accompanied by a dramatic change in mitochondrial morphology, characterized by fragmentation and impaired dynamics. This is consistent with prior reports that link PA‐induced mitochondrial fragmentation to apoptosis in neurons and astrocytes (Langley et al. 2020; Takuma et al. 2004; Kumar Jha et al. 2016).

Furthermore, the observed downregulation of MFN2 and phosphorylated DRP1 at Ser616 (p‐DRP1) suggests that PA at 100 µM may disrupt the mitochondrial fusion‐fission balance, potentially contributing to mitochondrial dysfunction during oligodendrocyte maturation. These alterations coincide with a loss in ROS‐producing capacity, suggesting advanced mitochondrial failure. The loss of the ability to maintain fusion‐fission balance, the absence of ROS production and the associated decrease in ΔΨm in the presence of PA 100 µM suggest that mitochondria become static and dysfunctional, eventually leading to apoptosis.

PA has been reported to induce mitochondrial stress and activate mitophagy as an early adaptive mechanism to maintain mitochondrial quality control (Tian et al. 2025; Jiang et al. 2020; Wu et al. 2015). Accordingly, the mitochondrial fragmentation and functional loss observed at PA 100 µM might indicate a transition from an initially compensatory quality‐control response to irreversible mitochondrial failure, where insufficient mitophagy may lead to the accumulation of dysfunctional mitochondria and activation of caspase‐dependent apoptosis.

Conversely, no lipotoxic PA concentrations, such as 25 µM, induced the differentiation of OPCs. This effect is supported by alterations in mitochondrial dynamics (1 d) favoring fusion, as indicated by increased MFN2 and decreased p‐DRP1[Ser616] levels, which mirror the energetic needs during early differentiation. Mitochondrial fusion is essential for OLs maturation which requires a high energy supply, provided by mitochondria, the main organelles involved in ATP production, to support biosynthesis for membrane production (Gil and Gama 2023). Several mitochondrial signaling pathways contribute to OPCs survival and myelination (Sun et al. 2018). Previous studies show that mitochondrial fusion predominates in the early maturation stage of OLs (Yazdankhah et al. 2021; Soares et al. 2024; Kirischuk et al. 1995). In more advanced stages of differentiation, however, a shift to mitochondrial fission becomes beneficial, facilitating metabolic adaptation and organelle distribution (Yazdankhah et al. 2021). Accordingly, we found that at a later point in the differentiation process of OLs (4 d), mitochondrial dynamics are altered with an increase in protein expression level of p‐DRP1[Ser616], which promotes mitochondrial fission.

In addition, PA 25 µM reduced intracellular ROS and induced nuclear translocation of Nrf2, with subsequent increase in its downstream antioxidant and metabolic targets. The activation of Nrf2 and PPARγ pathways not only preserves redox balance but also supports mitochondrial biogenesis and differentiation (De Nuccio et al. 2020; Paintlia et al. 2010). These transcriptional factors regulate the abundance of CD36, which plays a key role in the uptake of FA‐containing substrates (Grajchen et al. 2020; Ioghen et al. 2021). Cells use LDs as intracellular energy stores in the form of neutral lipids that can be mobilized to meet cellular energy demands. LDs release free FAs, which are distributed among subcellular organelles for energy production or membrane biogenesis. The increase in CD36 and concomitant reduction in lipid droplet accumulation might suggest a possible utilization of lipids for membrane biosynthesis, consistent with the metabolic needs of differentiating OLs.

In the CNS, lipid homeostasis is tightly linked to inflammation and demyelination. Dysregulated lipid metabolism has been implicated in the pathogenesis of MS, particularly in chronic lesions (Ladakis et al. 2024; Damiano et al. 2015) where impaired OPCs differentiation persists (Kuhlmann et al. 2008) and altered antioxidant responses in immune cells are associated with disease modulation (Rubino et al. 2021). Interestingly, epidemiological studies have shown a negative correlation between some dietary saturated fat intake, including palmitate, and MS progression, although confounding dietary and genetic factors complicate interpretation (Hon et al. 2009).

As shown in this study, low levels of PA are beneficial, particularly in supporting cell differentiation through activation of metabolic pathways involving PPARγ and CD36. Whether CD36 directly modulates OL maturation remains underexplored, but its role in lipid trafficking suggests it could serve as a key mediator of myelin biogenesis.

In addition, this study explored the effects of low‐dose PA on modulation of OPCs migration and cell cycle progression, processes essential for effective myelination and early steps to induce differentiation. Specifically, we observed a blockade of early maturation phases (migration and proliferation) and an increase in differentiation markers such as MBP and PLP (Dugas et al. 2006).

In line with these *in vitro* findings, the *ex vivo* experiments in hippocampal and cerebellar organotypic slices consistently showed that low‐dose PA treatment exerts neuroprotective actions under neuroinflammatory conditions, enhances developmental myelination and accelerates remyelination in a model of myelin damage and repair. These *ex vivo* data confirm that low, non‐lipotoxic concentrations of PA can exert beneficial effects in a 3D CNS tissue‐like environment, and indicate that the non toxic profile of PA observed in MO3.13 cells is preserved under pathophysiologically relevant demyelinating conditions. Although organotypic slice cultures do not fully recapitulate the complexity of *in vivo* demyelinating diseases, they represent an important intermediate level between simplified cell systems and whole‐animal models. Our combined *in vitro* and *ex vivo* results therefore support the existence of a disease relevant window of efficacy for PA at low concentrations, while highlighting the potential risks associated with higher doses (Doussau et al. 2017; Sacks et al. 2018).

Our findings reinforce the concept that lipid‐derived metabolic signals are integral physiological regulators of OPC behavior and myelin sheath formation. Alterations in lipid profiles, as observed in metabolomic studies of MS patients, may reflect disruptions in homeostatic mechanisms essential for oligodendroglial function (Ferreira et al. 2020). The biphasic response to PA underscores the complexities of lipid signaling in regulating OPC functions highlighting the physiological importance of metabolic balance in central nervous system health.

## 5 Conclusions

Our findings identify PA as a context and dose‐dependent modulator of oligodendrocyte function. High concentrations of PA (100 μM) are lipotoxic, inducing mitochondrial dysfunction and apoptosis, whereas low, non‐cytotoxic levels (25 μM) promote antioxidant responses, support OPC differentiation, and exert neuroprotective and pro‐myelinating/remyelinating effects in organotypic CNS tissue models. Further *in vivo* studies in demyelination models are warranted to validate these findings, assess their translational potential, clarify the contribution of neuron‐glia and glia‐glia interactions to PA's actions, and better define the therapeutic window and safety profile of PA dependent lipid‐modulating strategies to enhance remyelination and neuroprotection in MS and related disorders. Our study underlines the need to tailor dietary FA intake to support oligodendrocyte health and opens avenues for nutritional interventions aimed at promoting remyelination and neuroprotection in neurodegenerative conditions such as MS.

## Author Contributions

**Anna Palmiero:** formal analysis, investigation, data curation, writing – original draft. **Luca Pipicelli:** formal analysis, investigation, data curation, writing – original draft. **Giuliana La Rosa:** investigation, data curation. **Concetta Sozio:** investigation, data curation. **Carolina Punziano:** methodology, formal analysis. **Maddalena Raia:** methodology. **Raffaella Faraonio:** writing – review and editing, supervision. **Giovanna Vitolo:** investigation, data curation. **Mariarosaria Cammarota:** investigation, data curation. **Francesca Boscia:** conceptualization, investigation, data curation. **Ciro Menale:** conceptualization, data curation, writing – review and editing. **Mariarosaria Santillo:** conceptualization, investigation, data curation, writing – original draft, supervision. **Simona Damiano:** conceptualization, investigation, data curation, writing – original draft, writing. All authors have read and agreed to the published version of the manuscript.

## Ethics Statement

All animal procedures were conducted in accordance with the ARRIVE guidelines and the Guide for the Care and Use of Laboratory Animals (EU Directive 2010/63/EU). The experimental protocol was approved by the Animal Care and Use Committee of the University of Naples “Federico II” and by the Italian Ministry of Health (#515/2019‐PR). All efforts were made to minimize animal suffering and to reduce the number of animals used.

## Conflicts of Interest

The authors declare no conflicts of interest. The funders had not involvement in the study design; data collection, analysis, or interpretation; manuscript writing; or the decision to submit the work for publication.

## Data Availability

The data supporting the findings of this study are available within the article.
